# A Novel Network-Based Algorithm for Predicting Protein-Protein Interactions Using Gene Ontology

**DOI:** 10.3389/fmicb.2021.735329

**Published:** 2021-08-25

**Authors:** Lun Hu, Xiaojuan Wang, Yu-An Huang, Pengwei Hu, Zhu-Hong You

**Affiliations:** ^1^Xinjiang Technical Institute of Physics and Chemistry, Chinese Academy of Sciences, Ürümqi, China; ^2^School of Computer Science and Technology, Wuhan University of Technology, Wuhan, China; ^3^College of Computer Science and Software Engineering, Shenzhen University, Shenzhen, China; ^4^School of Computer Science, Northwestern Polytechnical University, Xi'an, China

**Keywords:** protein-protein interaction, prediction, network topology, gene ontology, modularity

## Abstract

Proteins are one of most significant components in living organism, and their main role in cells is to undertake various physiological functions by interacting with each other. Thus, the prediction of protein-protein interactions (PPIs) is crucial for understanding the molecular basis of biological processes, such as chronic infections. Given the fact that laboratory-based experiments are normally time-consuming and labor-intensive, computational prediction algorithms have become popular at present. However, few of them could simultaneously consider both the structural information of PPI networks and the biological information of proteins for an improved accuracy. To do so, we assume that the prior information of functional modules is known in advance and then simulate the generative process of a PPI network associated with the biological information of proteins, i.e., Gene Ontology, by using an established Bayesian model. In order to indicate to what extent two proteins are likely to interact with each other, we propose a novel scoring function by combining the membership distributions of proteins with network paths. Experimental results show that our algorithm has a promising performance in terms of several independent metrics when compared with state-of-the-art prediction algorithms, and also reveal that the consideration of modularity in PPI networks provides us an alternative, yet much more flexible, way to accurately predict PPIs.

## 1. Introduction

As one of the most common and indispensable molecules in cells, proteins are critical in regulating various biological processes observed in living organisms by interacting with other different proteins through protein-protein interactions (PPIs) (Hu et al., 2021a). Since PPIs are of great significance to undertake many physiological functions, there is a necessity for us to identify PPIs from cells in order to fully explore the cellular mechanism behind biological processes.

In the last decades, a large number of prediction methods have been developed to verify the interacting relationship between pairwise proteins, and they are divided into two categories, one is laboratory-based and the other is computational-based. The technologies in the former category include, but not limited to, yeast two-hybrid (Fields and Sternglanz, 1994), TAP-tagging (Ho et al., 2002), and protein chips (Zhu et al., 2001). They normally suffer the disadvantage of being time-consuming and labor-intensive, thus resulting in an inefficient identification of PPIs. To overcome these problems, attempts have been made to develop different computational algorithms for PPI prediction. In particular, computational algorithms mainly put their efforts on extracting useful features from the biological information of proteins, such as protein sequences (Zahiri et al., 2013; Hu and Chan, 2015), protein structures (Zhang et al., 2012; Mirabello and Wallner, 2017), and co-evolutionary profiles (Hsin Liu et al., 2013; Hu and Chan, 2017), that are able to explicitly represent the characteristics of proteins, and then solve the problem of PPI prediction as a binary classification problem. Though efficient, most of them are unable to handle the structural information of PPI networks for better performing the prediction task. Moreover, regarding the fact that the amount of PPI data have also increased significantly with the development of high-throughput technologies, studies have been conducted to develop various prediction algorithms that are able to complete the task of PPI prediction in a distributed manner (You et al., 2014; Hu et al., 2017).

As a recent attempt in network-based PPI prediction, L3 (Kovács et al., 2019) reckons that the traditional triadic closure principle is inappropriate for predicting PPIs from a given PPI network, as two proteins are more likely to interact if one of them is similar to the other's partners rather than sharing many common interacting partners. Experimental results demonstrate that L3 significantly outperforms existing link prediction methods when applied to solve the PPI prediction problem. Given two proteins, since L3 only considers their common interacting partners, the network paths involved are with the same length, i.e., 3. In this regard, L3 is incapable of determining the interaction between proteins that are far away from each other without any common neighbors. To address this problem, Wang et al. (2020) design a novel stochastic block model, namely PPISB, for predicting PPIs without specifying the length of network paths in advance. PPISB can capture the latent structural features of proteins in a PPI network, thus verifying whether two proteins interact with each other or not. However, a major concern for network-based algorithms is the quality of PPI networks. In particular, when composing a PPI network, the PPI data generated by high-throughput technology is characterized by high false-positive and false-negative rates, and accordingly the accuracy performance of network-based prediction algorithms is severely affected. Similar to L3 and PPISB, network-based distance Analysis can also be applied to predict lncRNA-miRNA Interactions (Zhang et al., 2021).

As has been pointed out by Hu et al. (2021b), proteins in the functional modules are densely connected with each other. In other words, for two proteins in the same functional module, their probability of being interacting should be considerably larger than those across different functional modules. Moreover, the neighboring relationship between molecules has also been verified to be useful for predicting their interactions (Liu et al., 2020). Hence, we believe that the performance of PPI prediction can be further improved by taking into account this motivation. In this work, we target to integrate the biological information of proteins, specifically Gene Ontology (GO), into a given PPI network, thus alleviating the negative influence of noise data. Motivated by the aforementioned intuition that proteins in the same functional module are more likely to interact with each other, we adopt an established Bayesian model proposed by Hu et al. (2020) to simulate the generative process of PPI networks together with associated GO information by assuming that the prior information of functional modules are known in advance. After that, a novel scoring function is designed to compute the interaction probability of two proteins according to their membership distributions and network paths. Following this pipeline, we develop a new algorithm, namely NGPM, to complete the task of PPI prediction. To evaluate the performance of NGPM, a series of extensive experiments have been conducted by comparing it with several state-of-the-art PPI prediction algorithms on five practical PPI networks collected from different species, and an in-depth discussion about experimental results is provided to demonstrate the superiority of NGPM in predicting PPIs.

The rest of this paper is organized as follows. In section 2, the details of NGPM are described. Experimental results are presented in section 3, following which we end with an in-depth discussion in section 4.

## 2. Materials and Methods

Given the fact that proteins interact with each other in cells to form functional modules, a single protein is possible to be involved in multiple protein complexes and thereby undertake different physiological functions. For a PPI network associated with GO information of proteins, we first assume that a total of *K* functional modules are existed and the details of generating such a PPI network is first presented by adopting the Bayesian model proposed by Hu et al. (2020). After that, we describe the complete procedure of NGPM.

### 2.1. Mathematical Preliminaries

A PPI network of interest is formally denoted as a four-element tuple *G* = {*V, A, X*, Λ}, where *V* = {*v*_*i*_}(1 ≤ *i* ≤ *n*_*V*_) is a set of all *n*_*V*_ proteins, *A* = [*A*_*ij*_] is a *n*_*V*_×*n*_*V*_ adjacency matrix where *A*_*i*_*j* = 1 if two proteins, i.e., *v*_*i*_ and *v*_*j*_, interact with each other and 0 otherwise, *X* = {*X*_*i*_}(1 ≤ *i* ≤ *n*_*V*_) consists of the GO information of proteins in *V*, and Λ = {Λ_*m*_}(1 ≤ *m* ≤ *n*_*V*_) denotes a set of total *n*_Λ_ GO categories that are available to be associated with proteins. Obviously, *A* and *X* describe *G* from the perspectives of network topology and GO, respectively. In this regard, an instance of *G* can thus be obtained if *A* and *X* are determined.

Regarding *X*, each element, i.e., *X*_*i*_ = {*x*_*ip*_}, denotes the set of GO annotations taken by *v*_*i*_ without considering GO categories, and the size of *X*_*i*_ is |*X*_*i*_|. The combination of *X*_*i*_ and Λ preserves the necessary details to sample the GO information for each protein. Assuming that Λ_*ip*_∈Λ is the GO category of *x*_*ip*_ and *dom*(Λ_*m*_) is a set of possible GO annotations in Λ_*m*_, we have *x*_*ip*_∈*dom*(Λ_*m*_) if Λ_*ip*_ = Λ_*m*_. The size of *dom*(Λ_*m*_) is denoted as |*dom*(Λ_*m*_)|.

To indicate the functional modules of proteins, we adopt a *n*_*V*_×1 vector, i.e., *C* = [*C*_*i*_](1 ≤ *i* ≤ *n*_*V*_, 1 ≤ *C*_*i*_ ≤ *K*), where *C*_*i*_ represents the functional module label of *v*_*i*_. Therefore, for an arbitrary protein, i.e., *v*_*i*_, its *C*_*i*_ is equal to *k* if it is in the *k*-th functional module.

### 2.2. Generating Functional Module Labels

For an arbitrary protein denoted as *v*_*i*_, its functional module label *C*_*i*_ is chosen from a Multinomial distribution, which is defined as (1).

(1) $$\begin{array}{r} p\left(C_{i}=k|\alpha \right)=\alpha_{k}\left(1\leq k\leq K\right) \end{array}$$

where α_*k*_ is the probability of a protein that is assigned to the *k*-th functional module and $\overset{K}{\underset{k=1}{\sum }}\alpha_{k}=1$. Instead of predetermining the value of each element in α, we consider α as a random variable and sample it by using a Dirichlet distribution with a parameter ζ.

### 2.3. Generating GO Information of Proteins

In order to completely retain the relationship between GO categories and their corresponding annotations, we sample the GO annotations of *v*_*i*_ with two steps. Specifically, to obtain *x*_*ip*_, we first choose its GO category, i.e., Λ_*ip*_, from a Multinomial distribution that is specific to the functional module of *v*_*i*_. Hence, we have

(2) $$\begin{array}{r} p\left(\Lambda_{ip}=\Lambda_{m}|\theta_{C_{i}}\right)=\theta_{C_{i}m}\left(1\leq m\leq n_{\Lambda }\right) \end{array}$$

In the above equation, θ_*C*_*i*__ is a *n*_Λ_-dimensional variable randomly selected from the Dirichlet distribution with a parameter λ_*C*_*i*__. As the subscript of λ_*C*_*i*__, *C*_*i*_ indicates that the probability distribution of λ_*C*_*i*__ is conditioned on the functional module label of *v*_*i*_.

**Figure 1:**
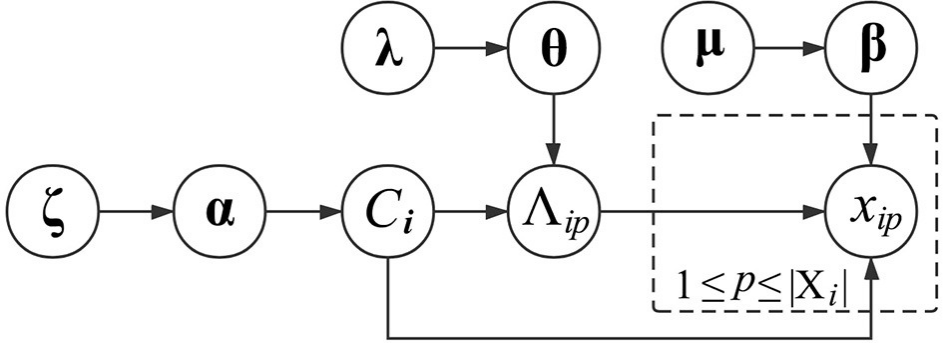
Graphical model representation of generating the GO information of proteins.

Once the GO category of *x*_*ip*_ is determined, the next step is to select the annotation of *x*_*ip*_ from the domain of Λ_*ip*_. Assuming that Λ_*ip*_ is actually the *m*−th category in Λ, i.e., Λ_*m*_, the value of *x*_*ip*_ is then sampled from a Multinomial distribution defined as:

(3) $$\begin{array}{r} p\left(x_{ip}=val_{mt}|\beta_{C_{i}m}\right)=\beta_{C_{i}m}^{t}\left(1\leq t\leq |dom\left(\Lambda_{m}\right)|\right) \end{array}$$

where *val*_*mt*_ is the *t*-th annotation in *dom*(Λ_*m*_). Regarding the subscripts *C*_*i*_ and *m*, their combination indicates that the Multinomial distribution of *x*_*ip*_ is specific to the functional module of *v*_*i*_ and the GO category Λ_*m*_. In other words, proteins in the same functional module share similar Multinomial distributions of GO annotations, which can differ across different GO categories or functional modules. To generate β_*C*_*i*_*m*_, we also place a Dirichlet distribution over it with a prior parameter μ_*C*_*i*_*m*_. The graphical presentation of generating the GO information of proteins is presented in Figure 1.

### 2.4. Generating PPIs

As mentioned before, we introduce *A* to represent the interaction relationships for all pairwise proteins in *G*. Hence, generating PPIs in a PPI network is identical to generate *A*. Following the observation that proteins in the same functional module are densely connected, the value of *A*_*ij*_ is dependent on a finite mixture of functional modules labels according to Stochastic BlockModel (Nowicki and Snijders, 2001).

Given two proteins, i.e., *v*_*i*_ and *v*_*j*_, the probability of *v*_*i*_ interacting with *v*_*j*_ follows a Multinomial distribution described below.

(4) $$\begin{array}{r} p\left({\text{A}}_{ij}=1|C_{i}=k,C_{j}=l,\varepsilon_{k}\right)=\varepsilon_{kl} \end{array}$$

**Figure 2:**
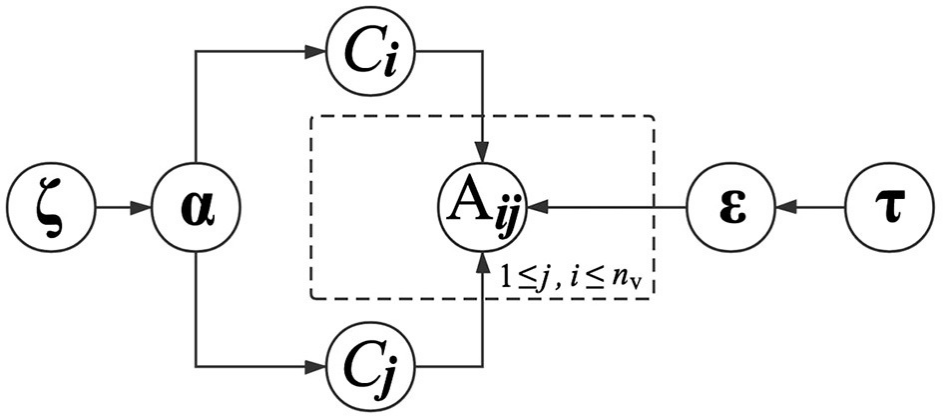
Graphical model representation of generating PPIs.

In the above equation, the parameter ε_*kl*_ is conditioned on the functional module labels of *v*_*i*_ and *v*_*j*_. The interaction probabilities between all pairs of functional modules are therefore parameterized by **ε**, which is a *K*×*K* matrix. With (4), proteins in the same functional modules present similar regularities when interacting with other proteins. Similarly, we also place a Dirichlet distribution with a prior parameter **τ**_*k*_ to determine **ε**_*k*_. The graphical presentation of generating PPIs is presented in Figure 2.

So far, the generative process of *G* is completed by the above generative process that involves several latent variables **α**, **θ**, **β**, and **ε**. Regarding the values of these variables, we also define corresponding prior parameters, i.e., **ζ**, **λ**, **μ**, and **τ**, to sample them in a Bayesian manner.

### 2.5. Bayesian Decision

According to the above generative process, a PPI network, i.e., *G*, is represented as a collection of proteins, PPIs and GO annotations. To indicate the functional module label of each protein, we need to compute the probability of each possible *C* conditioning on both *A* and *X*, and select the one with the maximum posterior probability as the optimal result. Hence, we can formulate an optimization problem as below.

(5) $$\begin{array}{r} Ĉ={\arg\max}_{C}p\left(C|A,X,\zeta ,\lambda ,\mu ,\tau \right) \end{array}$$

To address this problem, we apply the solution developed in Hu et al. (2020). Instead of explicitly determining *C*, this solution yields the optimal membership matrix, i.e., $\hat{\alpha }=\left[{\hat{\alpha }}_{ik}\right]$ to derive Ĉ. Specifically, for *v*_*i*_, its functional module label *C*_*i*_ is more likely to be equal to *k* if ${\hat{\alpha }}_{ik}$ is larger.

### 2.6. Computing Interaction Probability

To indicate to what extent two proteins are likely to interact, a scoring function is designed by taking into account their membership distributions and network paths simultaneously. The motivation of designing such a function is twofold. First of all, for two proteins, the probability of being grouped in the same functional module is larger if their membership distributions are more similar, and accordingly they are more likely to interact with each other. On the other hand, two proteins are less likely to interact if the network path connecting them is longer. Assuming that *L*_*v*_*i*_*v*_*j*__ is a set of all network paths connecting *v*_*i*_ and *v*_*j*_ in *G* and its size is |*L*_*v*_*i*_*v*_*j*__|, the scoring function is defined as below.

(6) $$\begin{array}{r} score\left(v_{i},v_{j}\right)=\overset{\left|L_{v_{i}v_{j}}\right|}{\underset{w=1}{\sum }}weight{\left(L_{w}\right)}^{decay\left(L_{w}\right)} \end{array}$$

In the above scoring function, *weight*(*L*_*w*_) evaluates the strength of *L*_*w*_ in terms of providing evidence to support the interaction between *v*_*i*_ and *v*_*j*_ and its definition is given as:

(7) $$\begin{array}{r} weight\left(L_{w}\right)=\overset{\left|L_{w}\right|}{\underset{z=1}{\prod }}{\hat{\alpha }}_{zk} \end{array}$$

where *k* is the value of *C*_*i*_, |*L*_*w*_| is the number of proteins in *L*_*w*_ and ${\hat{\alpha }}_{zk}$ is the membership over the *k*-th function module for the *z*-th protein along the path *L*_*w*_. Obviously, the value of *weight*(*L*_*w*_) is determined by the likelihood of being group in the function module of *v*_*i*_ for the remaining proteins in *L*_*w*_.

Regarding *decay*(*L*_*w*_), the motivation of introducing this term is that it is less likely to interact with each other if two proteins are located far away from each other in a given PPI network. Hence, the definition of *decay*(*L*_*w*_) is given by (8) where φ is the decay coefficient and usually set to be greater than or equal to 1. Since the value of *weight*(*L*_*w*_) ranges from 0 to 1, *decay*(*L*_*w*_) has a decay effect as an exponentiation. The longer the length of *L*_*w*_ is, the more obvious the decay effect of *decay*(*L*_*w*_) has. To achieve a balance between accuracy and time, the value of |*L*_*w*_| is set to be 3 in our experiments.

(8) $$\begin{array}{r} decay\left(L_{w}\right)=\varphi \times |L_{w}| \end{array}$$

For each pair of testing proteins, we propose a novel prediction algorithm, namely NGPM, to calculate their interacting probability. To begin with the prediction, NGPM ranks the scores of all pairs of proteins including known PPIs and newly predicted PPIs. Since a predicted PPI is more likely to be real if it is surrounded by more already known PPIs, a sliding window is set by NGPM by selecting the upper and lower 50 pairs of proteins as a reference for the given pair of proteins. NGPM calculates the percentage of known PPIs to all pairs of proteins in this window, and then regards this percentage as the interacting probability for the given pair of testing proteins.

## 3. Results

In this section, the performance of NGPM has been compared with several state-of-the-art prediction algorithms on five practical PPI networks and the evaluation metrics include Precision, Recall, f-measure, AUC, and PR-AUC.

### 3.1. Experimental Setup

In the experiments, five independent PPI networks collected from different species are used, and they are denoted as Yeast-Tong (Tong et al., 2004), Yeast-Krogan (Krogan et al., 2006), Human (Rolland et al., 2014; Kovács et al., 2019), *Escherichia coli* (*E. coli*) (Gagarinova et al., 2016), and Mouse (Malty et al., 2017) respectively. The first two datasets are obtained from the species of yeast, and the Human dataset is composed of three human PPI networks, i.e., HI-II-14 (Rolland et al., 2014), HI-III (Rolland et al., 2014), and HI-tested (Kovács et al., 2019). The rest datasets are generated from other species as indicated by their names. The statistics of all these PPI networks are presented in Table 1.

**Table 1:** Statistics of PPI networks used in the experiments.

**Dataset**	***N***	**E**	**k_*av*_**	**CC**
Yeast-Tong	964	3,846	7.98	0.15
Yeast-Krogan	2,708	7,123	5.26	0.19
Human	6,657	32,307	9.52	0.07
*E. coli*	312	5,108	32.74	0.19
Mouse	786	1,975	5.03	0.15

*N, number of proteins; E, number of PPIs; k_av_, average graph distance; CC, clustering coefficient*.

In the experiments, a five-fold cross-validation has been conducted to yield convincing results and the performance of NGPM is compared with that of ASNE (Liao et al., 2018) and L3 (Kovács et al., 2019) to demonstrate its superiority in PPI prediction. When generating the negative samples, i.e., non-interacting proteins, we adopt the same strategy as L3 for conducting a fair comparison. In particular, for each PPI network, a total of 244 pairs of non-adjacent proteins are randomly selected as negative samples and 100 pairs of them should contain at least one of proteins listed in the top 500 PPIs predicted by L3.

### 3.2. Parameter Sensitivity Analysis

As the most important parameter involved in NGPM, *K* determines the number of functional modules observed from a given PPI network. To investigate the sensitivity of NGPM to the change of *K*, we present the performance of NGPM by varying the value of *K* from 2 to 20 at a step size of 1. In doing so, we are able to determine the best value of *K* for each dataset.

**Figure 3:**
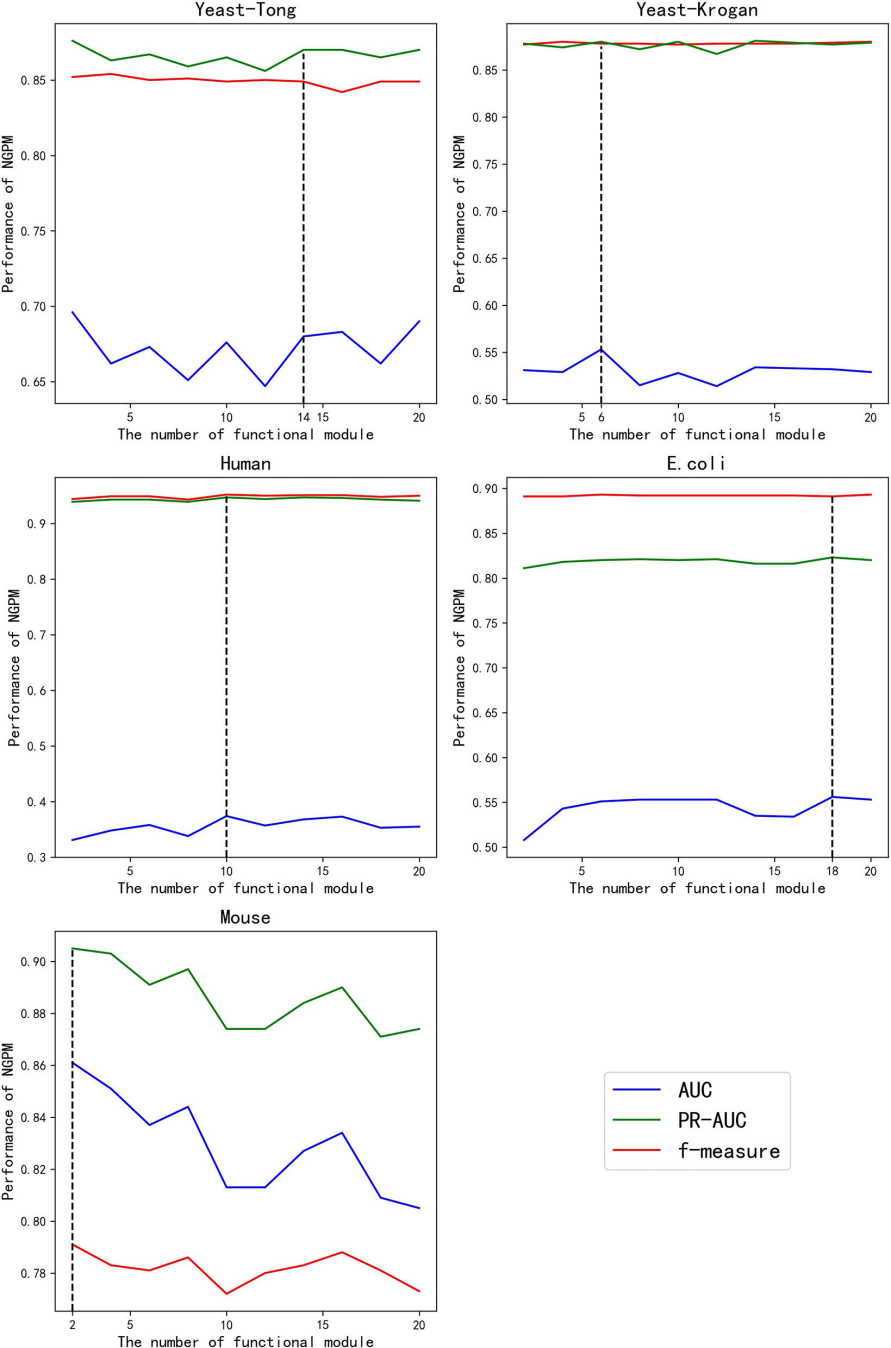
The performance of NGPM given different values of *K*.

Given different values of *K*, the performance of NGPM is presented in Figure 3. Fluctuations are observed for the AUC and PR curves, while the f-measure cures are more stable for all datasets except Mouse. A possible reason for that phenomenon is that f-measure is a harmonic mean of Precision and Recall. Since the increase in the score of *K* results in opposite changes of Precision and Recall, the fluctuation in the curve of f-measure is alleviated.

Among all kinds of curves in Figure 3, we also note that the robustness of NGPM in terms of AUC is the worst, as the AUC curves are more extensively fluctuated when compared with other curves. After investigating the experimental results, we find that the false-positive rates obtained by NGPM with different values of *K* are different, thus having a significant impact to the change of AUC curves. Another point worth noting is that the AUC curves are below the PR and f-measure curves for all datasets except Mouse. The reason for the unsatisfactory performance of AUC is due to the imbalance between positive and negative samples in the testing datasets.

According to Figure 3, the best values of *K* for Yeast-Tong, Yeast-Krogan, Human, *E. coli*, and Mouse are 14, 6, 10, 18, and 2, respectively. Hence, in the following experiments, we use the best performance of NGPM obtained by using these values for comparison.

### 3.3. Performance Comparison

**Figure 4:**
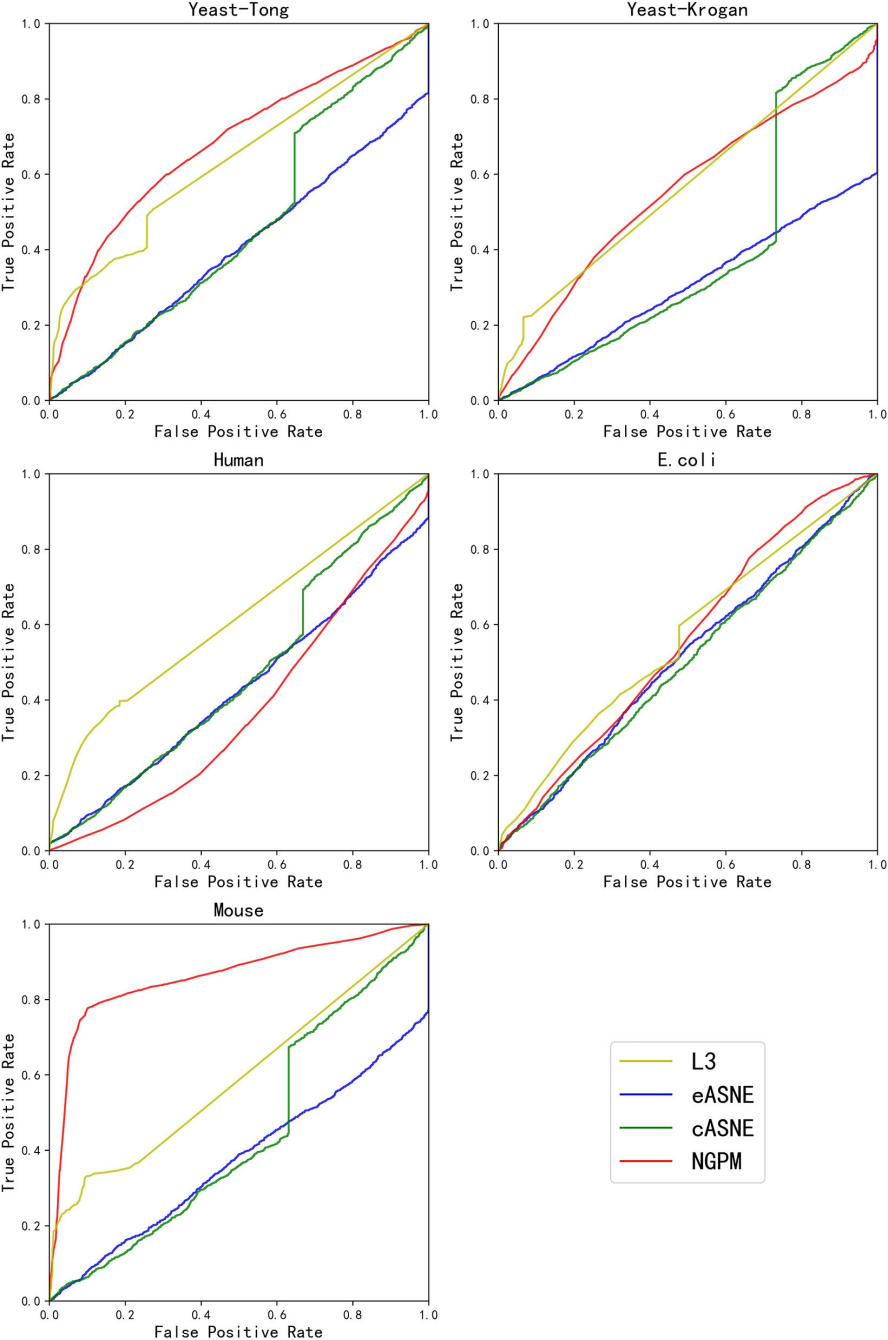
The ROC curves of L3, eASNE, cASNE, and NGPM.

**Figure 5:**
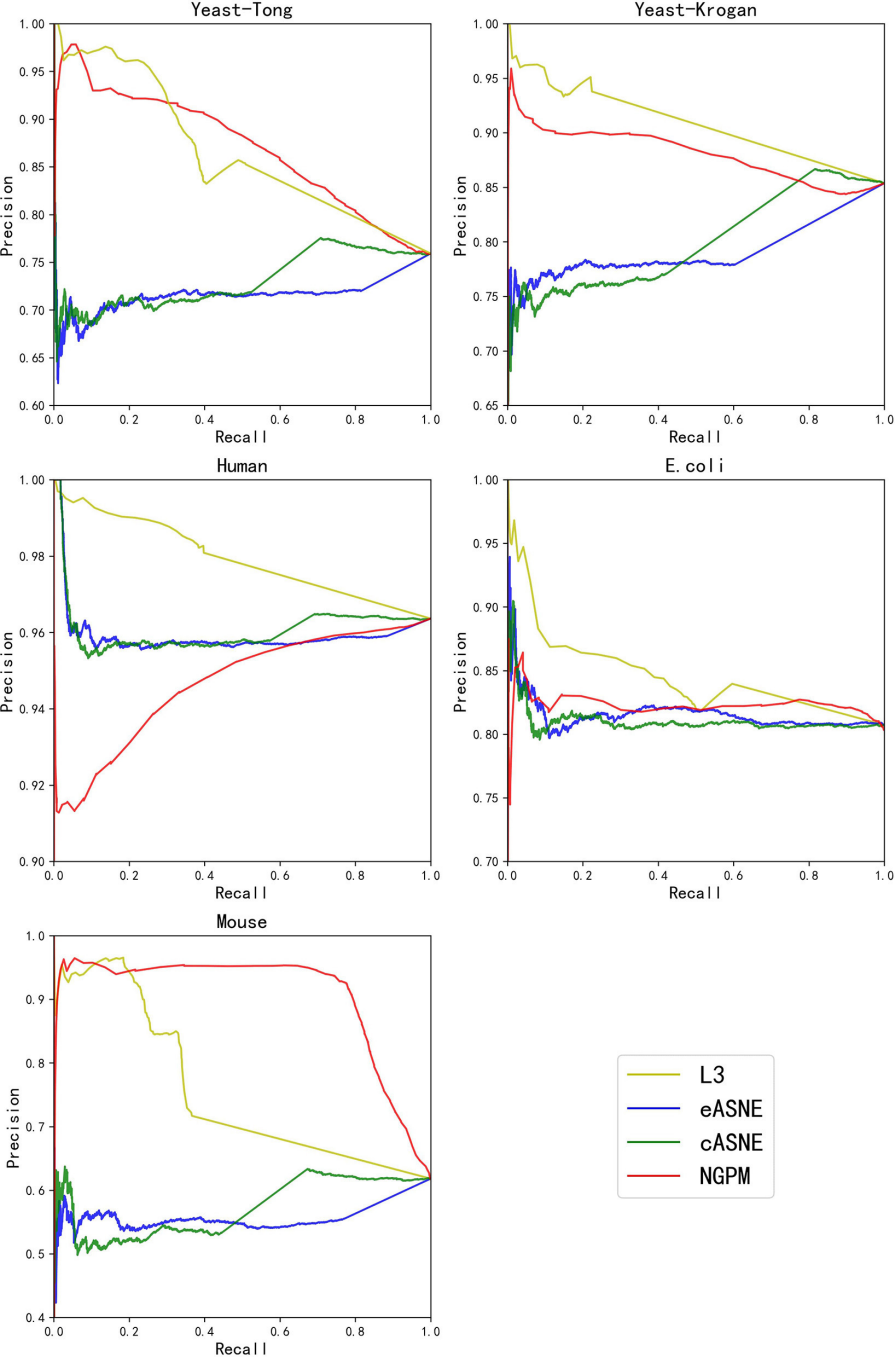
The PR curves of L3, eASNE, cASNE, and NGPM.

During the comparison, since ASNE can use different measurements to calculate the similarity between two proteins and determine their interacting probability accordingly, two most commonly used measurements including Euclidean similarity and cosine similarity are chosen in our experiments, and they are denoted as eASNE and cASNE, respectively. The results of performance comparison are shown in Figures 4, 5 and Table 2 where Figures 4, 5 show the ROC and PR curves of L3, NGPM, and ASNE obtained in each dataset, and Table 2 records the exact scores yielded by each prediction algorithm.

**Table 2:** The performance of models.

**Dataset**	**Model**	**f-measure**			**AUC**	**PR-AUC**
		**Precision**	**Recall**	**f-measure**
Yeast-Tong	L3	**0.84**	0.39	0.54	0.64	**0.87**
	eASNE	0.72	0.82	0.77	0.40	0.72
	cASNE	0.68	0.01	0.03	0.46	0.73
	NGPM	0.76	**0.96**	**0.85**	**0.68**	**0.87**
Yeast-Krogan	L3	**0.94**	0.19	0.31	**0.57**	**0.91**
	eASNE	0.78	0.61	0.68	0.30	0.79
	cASNE	0.69	0.01	0.01	0.39	0.80
	NGPM	0.85	**0.91**	**0.88**	0.55	0.89
Human	L3	0.98	0.39	0.56	**0.61**	**0.98**
	eASNE	0.96	0.85	0.90	0.43	0.96
	cASNE	**1.00**	0.02	0.03	0.47	0.96
	NGPM	0.96	**0.94**	**0.95**	0.37	0.95
*E. coli*	L3	0.82	0.53	0.64	**0.56**	**0.85**
	eASNE	0.81	1.00	0.89	0.52	0.82
	cASNE	**0.85**	0.02	0.04	0.50	0.81
	NGPM	0.81	**1.00**	**0.89**	**0.56**	0.82
Mouse	L3	**0.91**	0.23	0.37	0.59	0.75
	eASNE	0.55	0.77	0.65	0.37	0.56
	cASNE	0.60	0.02	0.04	0.44	0.57
	NGPM	0.68	**0.94**	**0.79**	**0.86**	**0.91**

*Best scores are bolded*.

When compared with ASNE, NGPM obtains a better performance on each metric across all the datasets except for Human and *E. coli*. On average, NGPM performs better by 6.28, 17.28, 12.08, 49.50, and 15.32% in terms of Precision, Recall, f-measure, AUC, and PR-AUC, respectively than eASNE while cASNE yields the worst performance among them. However, both NGPM and ASNE do not perform well on the Human dataset in terms of AUC. A main reason for that phenomenon is due to the serious imbalance between interacting and non-interacting proteins in the Human dataset. As mentioned before, the strategy of selecting negative samples in NGPM is as same as in L3, but it leads to the imbalance of interacting samples and non-interacting samples. Since the Human dataset is the largest one, it has more than 30,000 positive samples, while the negative sample is only 244. Thus it suffers the disadvantage of imbalance seriously and smaller AUC scores are obtained by all algorithms when compared with the other datasets.

**Figure 6:**
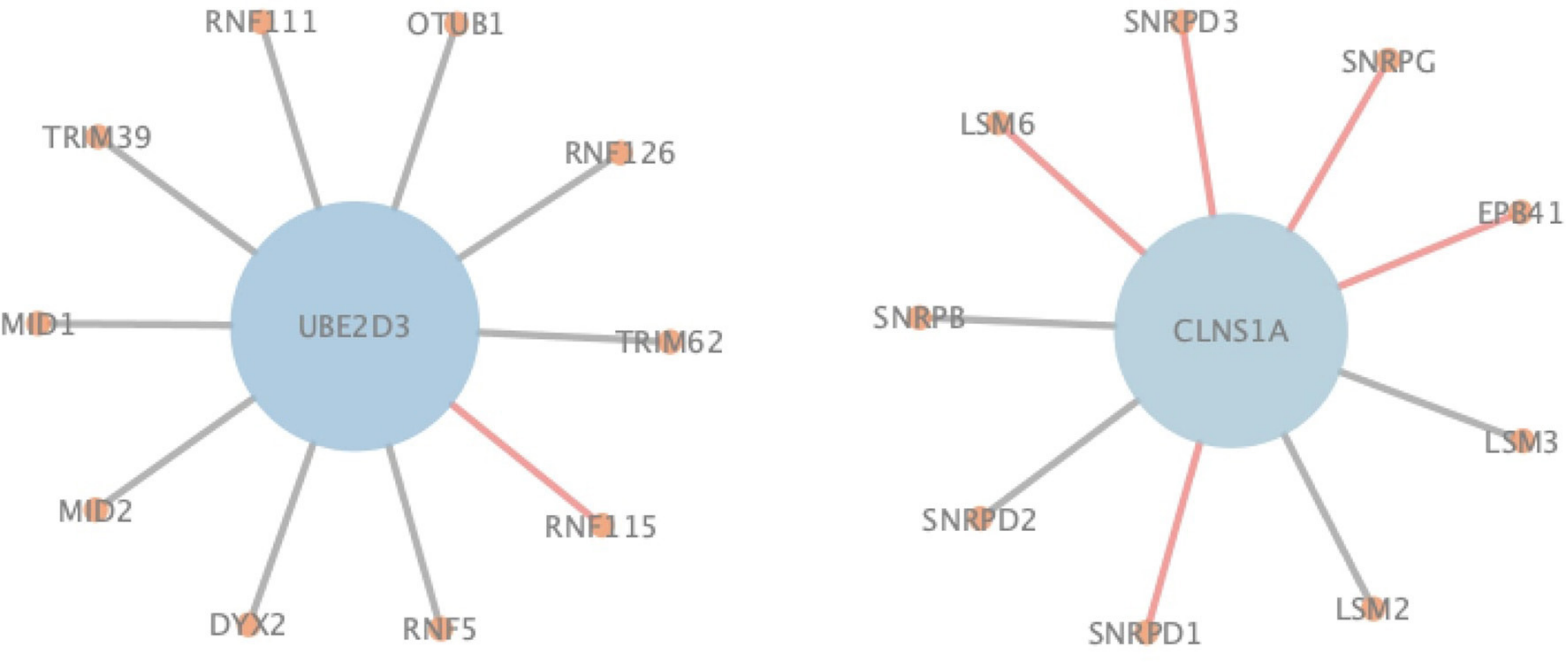
An illustration of PPIs correctly identified by NGPM in the Human dataset.

In order to more specifically illustrate the advantage of NGPM compared to ASNE in PPI prediction, we take the prediction results of NGPM on the Human dataset as an example. In particular, the nodes in Figure 6 represent proteins, and an edge connecting two nodes represents the interaction between them. Regarding the two proteins UBE2D3 and CLNS1A, they are classified as the negative sample in the testing dataset and thus there is no edge between them. However, ASNE predicts that they can interact with a probability as high as 0.76, thus leading to a wrong conclusion. NGPM accurately predicts the true relationship between UBE2D3 and CLNS1A. In the prediction result of NGPM, the interacting probability between these two proteins is < 0.4. Hence, NGPM is believed to be more reliable than ASNE when predicting PPIs. In addition, PPIs indicated by red lines are all successfully predicted by NGPM but incorrectly predicted by ASNE. These interactions have been verified by the BioGRID database (Chatr-Aryamontri et al., 2017) and can provide help for understanding the biological processes in the cell. Among them, CLNS1A, SNRPD1, EPB41, SNRPG, SNRPD3, and LSM6 are all important components of the cytoplasm, they can form protein complexes together. It is for this reason that NGPM is able to provide a precise prediction result for these proteins. Besides, all the proteins except EPB41 can participate in the process of RNA molecular interaction. Proteins UBE2D3 and RNF115 can also add ubiquitin groups to the proteins in cells to help them form ubiquitin chains, so that they can complete the catalysis of the ubiquitin reaction due to the interaction between them.

**Figure 7:**
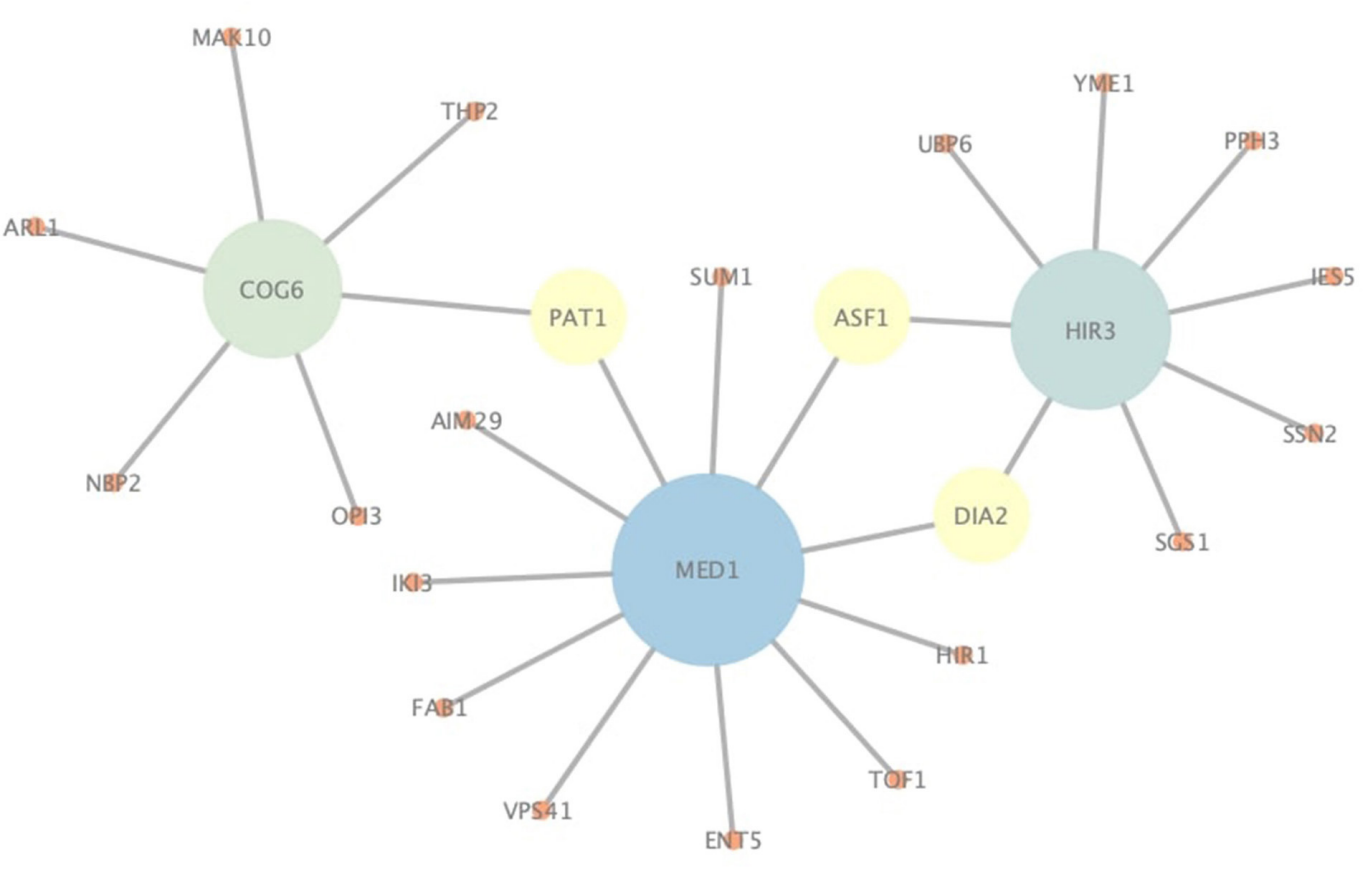
An illustration of novel PPIs identified by NGPM in Yeast-Tong dataset.

In addition to correctly predict known PPIs, NGPM is also capable of predicting novel PPIs that are not found in the testing dataset. Since NGPM allows each protein to be associated with a membership distribution and also finds the path between two proteins, the interacting probability can be determined by NGPM for any pair of proteins in a PPI network given such information. As indicated by Figure 7, several pairs of proteins extracted from the Yeast-Tong dataset are presented. PPIs represented by the edges are novel PPIs predicted by NGPM and these interactions have been confirmed by BioGRID database (Chatr-Aryamontri et al., 2017). In this regard, the ability of NGPM in predicting novel PPIs could thus be verified.

In order to verify whether NGPM can effectively eliminate the negative impact imposed by noise data such as false positives and false negatives after combining gene ontology and network topology, we compare the performance of NGPM on five PPI network with L3. From Table 2, NGPM obtains the best Recall and f-measure scores on all datasets. Specifically, when compared with L3, the performance of NGPM is better by 174.57, 79.42, 1.68, and 1.83% in terms of Recall, f-measure, AUC, and PR-AUC, respectively, and hence NGPM can reduce the negative impact caused by the noise data for PPI prediction. However, NGPM does not achieve the best performance on Precision, there are several reasons for this phenomenon. First of all The performance of NGPM is constrained by the existence of network paths. If there is no path between two proteins, NGPM can not predict the interaction between them and hence it will consider their interacting probability as 0. In doing so, a part of PPIs in the testing dataset are able to be predicted as non-interacting protein pairs, thus increasing the false negatives in the prediction result. Secondly, when predicting the interacting probability for proteins pairs, the longest path is set to be 3 in experiments, which is constrained by the computational efficiency of NGPM. A longer path will consume more time and we may be unable to obtain the prediction result after an acceptable period. Although the longer a path is, the less impact it has on determining the interacting probability between two proteins and consequently some PPIs are falsely predicted by NGPM. In this regard, the number of false positive samples obtained by NGPM is larger than the other algorithms, thus reducing the prediction accuracy of NGPM.

## 4. Discussion

In this paper, an efficient network-based prediction algorithm, namely NGPM, is proposed to predict PPIs by additionally considering the GO information of protein. The motivation behind NGPM is to make use of the property of functional modularity observed in PPI networks and also to combine the GO knowledge to alleviate the negative impact imposed by the noise data. Hence, by simulating the generative process of a PPI network, NGPM is able to incorporate these two kinds of information and optimize the membership distributions of proteins over functional modules. After that, a new scoring function is then designed to compute the interacting probability between two proteins. Experimental results have demonstrated that NGPM could better solve the prediction problem of PPIs as it yields a superior performance in terms of several independent metrics when compared with state-of-the-art prediction algorithms. In this regard, the novel PPIs predicted by NGPM may probably missed due to the constraints of laboratory experiments.

Several reasons can be summarized to explain the promising accuracy of NGPM. First of all, for a given protein, the modularity property of PPI networks allows NGPM to search potential interacting partners in a more accurate range, as proteins in the same functional module are more likely to interact with each other. However, there is no such a prior knowledge about the existence of functional modules in a PPI network before PPI prediction. By assuming the existence of total *K* functional modules embedded in a given PPI network, NGPM combines both network structure and GO to simulate the generative process of this network and then adopts an efficient solution to infer the membership distributions of proteins over functional modules. In doing so, the accuracy of PPI prediction can be improved. Secondly, to indicate how likely two proteins interact with each other, a novel scoring function is specifically designed by taking into account both network paths and membership distributions of proteins. It is also meaningful from a biological view. In particular, two proteins are more likely to interact with each other if they share many common interacting partners and are grouped into the same functional module together with these partners. Lastly, unlike conventional PPI prediction algorithms, NGPM does not rely on the selection of classifiers nor the generation of negative samples, thus making its performance more robust. One should note that the strategy of generating negative samples we describe in section 3.1 is only used for testing rather than training.

In addition to GO, there are also other kinds of biological information that can be used to characterize proteins. It is possible for NGPM to incorporate these biological information. Specifically, when generating the GO information of proteins, NGPM adopts different Multinomial distributions to sample the GO category and corresponding annotations. Hence, given a particular kind of biological information, we are able to incorporate it into NGPM if it can be represented as a set of attribute values taken by proteins.

Regarding future work, we would like to unfold it from three aspects. Firstly, since the longest length of paths used in (6) affects the performance of NGPM in some ways and we currently set it as 3 in the experiments, we intend to release this constraint by allowing NPGM to consider more path information. However, the increase in the longest length of paths could result in a consequence that more time will be taken by NPGM. Furthermore, there are many variational parameters that have to be optimized. The increase in the scale of PPI networks will obviously take more time to optimize these variational parameters. Hence, the current version of NGPM is not applicable for large-scale PPI prediction. To overcome this limitation, we would like to develop a distributed version of NGPM by following the MapReduce framework. Furthermore, regarding *K*, we have performed several trials to find its best value and thus we are also interested in providing a simpler, yet effective, strategy to determine its value. Lastly, since self-supervised pre-training has proven beneficial for many computer vision tasks, we would like to explore the possibility of pre-training NGPM on a different dataset when predicting PPIs.

## Data Availability Statement

Publicly available datasets were analyzed in this study. This data can be found at: https://gitee.com/allenv5/NGPM.

## Author Contributions

LH conceived of the study and drafted the manuscript. XW implemented the algorithms and carried out the experiments. LH, PH, and Z-HY conceived of the study, participated in its design and coordination, and helped to draft the manuscript. XW and Y-AH performed the statistical analysis. All authors read and approved the final manuscript.

## Conflict of Interest

The authors declare that the research was conducted in the absence of any commercial or financial relationships that could be construed as a potential conflict of interest.

## Publisher's Note

All claims expressed in this article are solely those of the authors and do not necessarily represent those of their affiliated organizations, or those of the publisher, the editors and the reviewers. Any product that may be evaluated in this article, or claim that may be made by its manufacturer, is not guaranteed or endorsed by the publisher.
